# Moving beyond Green: Exploring the Relationship of Environment Type and Indicators of Perceived Environmental Quality on Emotional Well-Being following Group Walks

**DOI:** 10.3390/ijerph120100106

**Published:** 2014-12-23

**Authors:** Melissa R. Marselle, Katherine N. Irvine, Altea Lorenzo-Arribas, Sara L. Warber

**Affiliations:** 1 Department of Psychology, Edge Hill University, St. Helens Road, Ormskirk, L39 4QP, UK; 2 Social, Economic and Geographical Sciences Research Group, The James Hutton Institute, Craigiebuckler, Aberdeen, AB15 8QH, UK; E-Mail: katherine.irvine@hutton.ac.uk; 3 Institute of Energy and Sustainable Development, De Montfort University, Leicester, LE1 9BH, UK; 4 BioSS—Biomathematics and Statistics Scotland, Craigiebuckler, Aberdeen, AB15 8QH, UK; E-Mail: altea.lorenzo-arribas@bioss.ac.uk; 5 Department of Family Medicine, University of Michigan Medical School, Ann Arbor, MI 48104, USA; E-Mail: swarber@umich.edu

**Keywords:** emotional well-being, perceived restorativeness, biodiversity, attention restoration theory, environmental quality indicators, green exercise, group walks

## Abstract

Against the backdrop of increasing interest in the relationship between Nature and health, this study examined the effect of perceived environment type and indicators of perceived environmental quality on short-term emotional well-being following outdoor group walks. Participants (*n* = 127) of a national group walk program completed pre- and post-walk questionnaires for each walk attended (*n* = 1009) within a 13-week study period. Multilevel linear modelling was used to examine the main and moderation effects. To isolate the environmental from the physical activity elements, analyses controlled for walk duration and perceived intensity. Analyses revealed that perceived restorativeness and perceived walk intensity predicted greater positive affect and happiness following an outdoor group walk. Perceived restorativeness and perceived bird biodiversity predicted post-walk negative affect. Perceived restorativeness moderated the relationship between perceived naturalness and positive affect. Results suggest that restorative quality of an environment may be an important element for enhancing well-being, and that perceived restorativeness and naturalness of an environment may interact to amplify positive affect. These findings highlight the importance of further research on the contribution of environment type and quality on well-being, and the need to control for effects of physical activity in green exercise research.

## 1. Introduction

For centuries, people have used the natural environment to enhance their health and well-being [1]. Empirical research has found that interaction with Nature is associated with better mental health and well-being [2,3,4,5,6], positive emotions [7,8,9,10], and attention [8,11,12], as well as reduced (physiological or perceived) stress [4,5,6,8,13,14,15,16,17,18]. Yet the natural environment is often “treated as uniform” [19] (p. 48) as studies commonly compare broad urban and natural environment categories [7,8,20] or analyze the amount of, or proximity to, Nature [2,3,21,22,23]. There have been calls to go “beyond the green” to investigate the contribution different types and qualities of natural environments have on well-being [24,25,26,27,28,29,30,31,32,33,34]. Specifically, Thompson Coon *et al.* [26] suggest “future studies might consider the impact of the perceived quality of the environment on mental and physical wellbeing outcomes.” (p. 1771). 

“Quality” is often discussed in terms of the “aesthetics or attractiveness” of the natural environment [35] (p. 27). Many of the indicators of environmental quality pertain to use, such as accessibility, maintenance, perceived safety, presence of amenities or absence of litter [27,29,35,36,37]. Recently, a broader set of environmental quality indicators have begun to be acknowledged and researched, such as: biodiversity [19,38,39,40,41,42], naturalness [36,43], and perceived restorativeness [33,43].

Despite the predominate focus in the literature to test exemplars of natural and urban environments, recent research has begun to investigate the influence different types and qualities of natural environments have on health and well-being. The following will review the previous literature that has moved beyond the green to investigate the effect on well-being from different environment types and indicators of perceived environmental quality—specifically perceived naturalness, biodiversity and restorativeness. 

### 1.1. Types of Natural Environments 

Not all green spaces have an equal impact on well-being; some types of natural environments may have more of an effect on well-being than others. For example, de Vries *et al.* [2] found that the amount of agricultural green space in one’s neighbourhood was associated with greater physical and mental health, but the amount of urban green space, forest, or “nature areas” (p. 1722) in the neighbourhood had no effect [2]. Similarly, living near to coastal environments have been shown to have an effect on positive mental health—over and above the effects of green space [44,45]. 

Use of specific types of natural environments for physical exercise has also been shown to have a differential effect on health and well-being. Exercise near a beach or river may have greater improvements in self-esteem and mood than exercising in urban green space, farmland and woodland environments [46]. Walking alone in a maintained forest was associated with greater positive affect and less negative affect, compared to walking alone in an unmaintained forest [47]. Marselle *et al.* [48] investigated the effect different environment types have on well-being and found that walking with others in farmland and green corridor environments were associated with less negative affect and perceived stress than walking with others in urban environments, whilst natural and semi-natural, urban green space, or coastal environments had no effect. 

### 1.2. Perceived Naturalness 

How natural an environment is perceived to be is an important predictor of well-being. People express greater positive affect and happiness in natural environments than in urban or indoor environments [7]. Environments perceived as “more natural” (e.g., forest, woodland, or valley) have been associated with greater psychological well-being than “less natural” environments (e.g., parks, gardens, or farmland) [9]. Perceived naturalness was a significant predictor of anxiety following a bout of green exercise; the more natural an environment is perceived, the larger the reductions in anxiety [49]. However, van den Berg *et al.* [50] found perceived naturalness of an environment had no influence on restoration of emotional well-being following a scary movie.

### 1.3. Perceived Biodiversity 

Biodiversity may be a useful environmental quality indicator for investigating the health and well-being impacts of natural environments [39]. The level of objective biodiversity in the environment has been shown to have a positive influence on improved health [28,39,51], psychological well-being [40] and positive emotions [41]. Our review here focuses on perceived biodiversity—an individual’s assessment of the species richness in an environment [19,39]. People have a general belief that the perceived biodiversity of flowers, birds, and trees in an urban park improves their well-being [42]. In their *in-situ* survey in riparian green space, Dallimer *et al.* [19] found psychological well-being was positively correlated with the number of bird, butterfly and plant species perceived in the environment. As investigations of biodiversity and well-being are a nascent research area, further research is needed to clarify the relationship between biodiversity and emotional well-being. 

### 1.4. Perceived Restorativeness

Examining the perceived restorative quality of an environment is another way to “move beyond green” in analyses of Nature and health. Attention Restoration Theory (ART) posits that certain environments can facilitate restoration of one’s ability to direct attention or concentrate [52,53]. Theorized qualities of a restorative environment include: being away, fascination, coherence, and compatibility [52,53]. A restorative environment requires psychological and physical distance from tasks, thoughts, or environments which require directed attention (*being away*). Fascinating stimuli are required to attract effortless, involuntary attention, which allows for the rest and restoration of directed attention (*fascination*). Fascination can be sustained if the stimuli are organized in a coherent way and rich enough to foster the perception of being in a whole other world (*coherence*). The theory acknowledges that a fit between the environmental setting and one’s purposes and inclinations is required for restoration; a compatible environment allows one to carry out his or her activities without struggle (*compatibility*). Natural environments are theorized to be well endowed with these four restorative qualities [52,53]. 

Quantitative measures have been created to assess the perceived restorativeness of an environment, based on the four qualities described by ART. These measures have been positively correlated with greater emotional well-being in general [54], and positive affect in particular [55]. Specific examination of the restorative quality of “fascination” found it was correlated with greater positive affect, but non-significantly related to negative affect [56]. 

### 1.5. Perceived Restorativeness as a Moderator

Environmental types and qualities do not occur in isolation; an environment can be experienced and assessed for its type, as well as its naturalness, biodiversity and restorative quality. For example, an urban green space could have a low level of perceived naturalness, moderate levels of perceived biodiversity and high perceived restorativeness. Similarly, a biodiverse environment can also be assessed for its naturalness, restorativeness, and environment type. 

While there are multiple ways in which one could conceptualise the relationship between the environment and well-being, in this paper we specifically focus on how these environmental characteristics might interact with one another to influence emotional well-being. Interaction is also known as moderation [57]. Moderators *qualify* environment-behaviour relationships [58]; they can answer *when* the external environment will effect well-being—and when it will not [57]. Moderation analyses of the relationship between environment and well-being have been called for by researchers [59,60]. Whilst previous studies have investigated gender [56], social interaction [61], activity type [62], and the type of urbanity surrounding a natural area [63] as moderators, few studies investigate an interaction between perceived environmental type and/or environmental qualities, and well-being. 

In the current study, we investigate whether perceived restorativeness would moderate the relationship between perceived type, naturalness, or biodiversity of an environment, and emotional well-being. ART implies that perceived restorative quality may interact with a natural environment to influence restorative outcomes. In other words, it is possible that a natural environment with high perceived restorative quality may engender greater restoration than a natural environment with low restorative quality. Hartig *et al.*’s [55] analysis of perceived restorativeness and emotional wellbeing lends some support for this argument. Whilst not formally testing for moderation, study 1 found the relationship between positive affect and perceived restorativeness differed by the restorative quality of an outdoor environment. Natural and built outdoor environments *a priori* expected to be high in restorative potential, had significant correlations between positive affect and perceived restorativeness, whereas those environments expected to be low in restorative potential demonstrated no significant relationship between positive affect and perceived restorativeness. Thus, environment and restorative quality give the appearance of having interacted, suggesting perceived restorativeness may effect when an outdoor environment influences emotional well-being and when it does not. In a study by Gonzalez *et al.* [64] the authors specifically investigated whether the restorative qualities of “being away” and “fascination” would moderate the effect of a therapeutic horticulture intervention on depression. Being away and fascination were measured as an average across multiple measurements, one of which included the respondent’s home. The authors found the overall level of “being away” moderated the change in depression of the therapeutic horticulture intervention, but ‘fascination’ did not. In other words, participating in the intervention was associated with greater decline in depression, among those who experienced a high level of “being away” in two environments (*i.e.*, home and the horticulture intervention setting). Due to the limited research, we believe there is scope to investigate whether perceived restorative quality interacts with perceived environmental type, naturalness or biodiversity to amplify well-being. 

### 1.6. Walk Characteristics—Walk Duration and Intensity

Physical exercise itself can improve mood [65,66,67]. As such, it is important to isolate its effect from the natural environment when studying green exercise [16,32,68]. Duration and intensity of physical activity both have been shown to increase post-exercise positive affect [69]. In this study, we measured duration and perceived intensity of the group walk to examine their independent influence on emotional well-being following an outdoor group walk.

### 1.7. Study Aims

The first aim of this study was to explore the health benefits of Nature beyond a “green” environment and investigate the effect of environment type and indicators of perceived environmental quality (*i.e.*, naturalness, biodiversity, restorativeness) on emotional well-being following an outdoor group walk. Characteristics of the group walk—duration and intensity—were assessed to understand their independent relationship to emotional well-being. The second aim of this study was to investigate whether perceived restorative quality of an environment moderates the effect of perceived environment type or perceived environmental quality on emotional well-being. To our knowledge, the interaction of perceived restorative quality with perceived environment type, naturalness, and biodiversity are heretofore unknown and such moderation analyses are unique. 

## 2. Methods 

### 2.1. Participants

**Figure 1:**
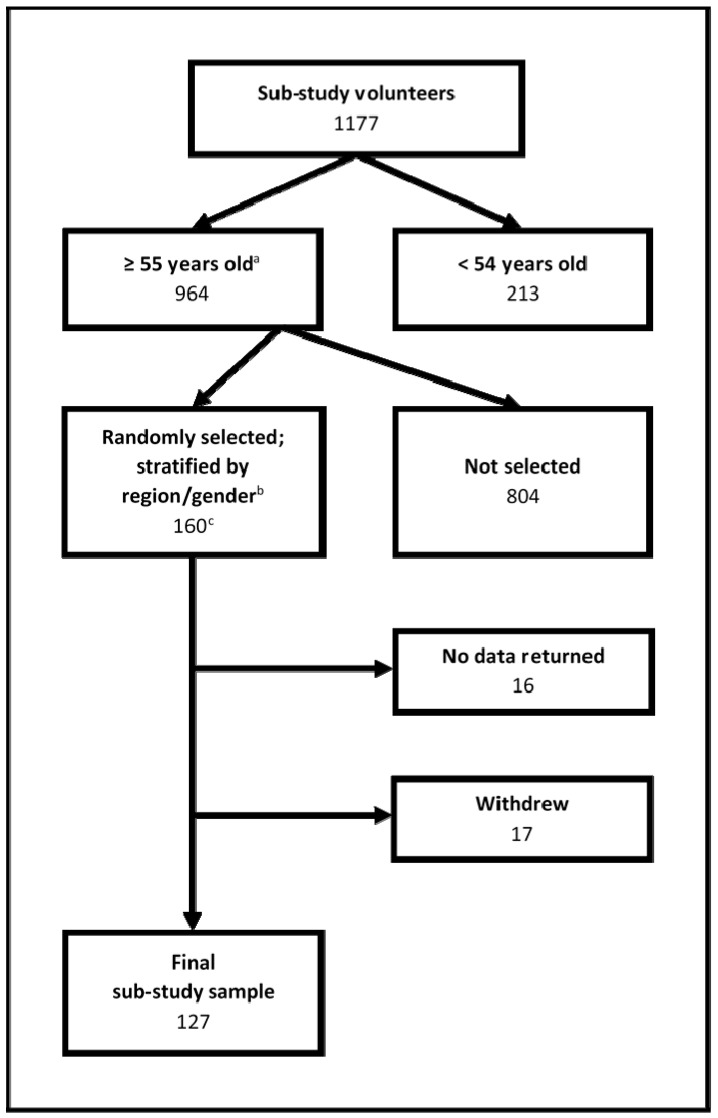
Steps in composing the sub-study sample beginning with participants from the main research study who volunteered to take part.

^a^ Selected to reflect Walking for Health demographics; ^b^Equal numbers of men and women for each region in England; ^c^Number determined by resource constraints.

Participants were recruited from a larger study investigating the well-being benefits of Walking for Health (WfH) [6], a national group walk program which provides free, short, led health walks throughout England [70]. Figure 1 details the participant flow. Over one-thousand participants of the main study volunteered to take part in this sub-study. Inclusion criteria was restricted to individuals aged 55 years or older to reflect the age demographic of the WfH population [71,72] and the main study sample [6]. One hundred and sixty individuals were randomly selected using a stratified (by English region and gender) sample. Information about age and gender of participants, and English region in which their WfH walk took place were collected through the main study. Thirty-three participants did not take part in the sub-study (see Figure 1). In total, 127 participants took part in this sub-study. The majority of participants were female (55.5%), and aged either 55–64 years (44.1%) or 65–74 years (45.5%).

### 2.2. Procedure

**Figure 2:**
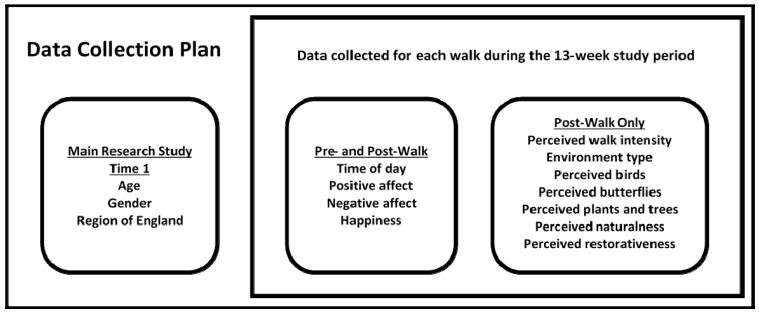
Data collection plan.

Note: Items from “Main Research Study Time 1” were collected via online questionnaires as part of the main study; information provided at “Pre-Walk” and “Post-Walk” were collected on paper-based questionnaires returned via mail. The variable “Duration of WfH walk” was calculated from Pre-Walk Start time and Post-Walk Finish time for use in analysis.

Participants completed a questionnaire for each WfH group walk attended within a 13-week study period (22 August to 14 November 2011). This 13-week period was the “intervention” for the main research study [6,73]. The two-page questionnaire contained a section to be completed immediately before, and a section to be completed immediately after, the walk. Figure 2 details the data collection plan. The date on which a walk took place was not collected on the questionnaire.

A “participant pack” containing a consent form, study instructions, 12 questionnaires (Additional questionnaires were available upon request from the first author if a participant took more than 12 walks during the 13-week study period.), and 13 self-addressed, stamped return envelopes was mailed to each participant prior to the start of the sub-study. Participants returned their signed consent form and completed questionnaires in the provided return envelopes. The study was approved by De Montfort University’s Human Research Ethics committee. A prize draw of £150 worth of shopping vouchers was provided as incentive for participation. 

### 2.3. Measures

#### 2.3.1. Outcome Variables

Emotional well-being—a form of subjective well-being concerned with hedonic experiences of happiness, pleasure and enjoyment [74,75]—was selected as the outcome of interest. Emotional well-being has a demonstrated impact on long-term health [76,77], and is one of the most common outcomes considered in studies of Nature and health [25,26,78]. It is often measured as the presence of positive feelings and the absence of negative feelings [75], and/or life satisfaction [79]. Consistent with previous research [7,8], we measured emotional well-being as positive affect, negative affect, and happiness. 

*Positive Affect* and *Negative Affect* were measured with the Positive and Negative Affect Schedule (PANAS) [80]. Before and after the group walk, participants rated how they felt “now” on 10 positive and 10 negative emotions using a 5-point scale (1 = *very slightly or not at all*; 5 = *extremely*). For each sub-scale, total scores range from 10 to 50; higher scores demonstrate greater positive or negative affect. The PANAS has been used in previous Nature and health studies [11,48,81,82]. *Happiness* was assessed before and after the group walk with a single item, 11-point scale (0 = *Not happy*; 10 = *Very happy*) [83]. Single-item happiness scales have been used in studies of restorative environments [7,8] and Nature and health [84,85].

#### 2.3.2. Predictor Variables

##### Environment Type

*Environment type* for a WfH group walk was assessed with the question: “*What type of environment did you walk in*?” Participants selected one response from a list of 10 categories that best described that environment. Environment types were reduced to seven categories based on previous research [48]. 

##### Indicators of Perceived Environmental Quality

*Perceived naturalness* of the environment was assessed with a single item, 7-point semantic differential scale (1 = *natural*; 7 = *artificial*). The item has been used in previous green exercise [49] and restorative environments [50] studies. This variable was inversely recoded (1 = *artificial*; 7 = *natural*) for the analysis. 

*Perceived biodiversity* was assessed with three items, in which participants were asked to indicate about how many different types of birds, butterflies, and plants and trees were in the environment. Items and response categories were based on previous research [40], with the addition of the initial response option of zero (Birds: 1 = 0; 2 = l–4 types; 3 = 5–14 types; 4 = 15–30 types; 5 = more than 30 different types; Butterflies: 1 = 0; 2 = 1–4 types; 3 = 5–9 types; 4 = 10–20 types, 5 = more than 20 different types; Plants and trees: 1 = 0; 2 = 1–9 types; 3 = 10–99 types; 4 = 100–300 types; 5 = more than 300 types). Due to low response frequencies for the zero response option, the two lowest response categories were combined for each perceived biodiversity variable; this aligned the response categories with those used by Fuller *et al.* [40].

*Perceived restorativeness* was measured using the 16-item Perceived Restorativeness Scale (PRS) [55,86]. Participants assessed the extent to which each statement reflected their experience of the environment on a 7-point scale (0 = *not at all*; 6 = *completely*). The items were placed in random order. Resulting scores range from 0 to 96; higher scores indicate greater perceived restorative quality. The PRS can discriminate between different types of natural environments [13,87] and has been used in a previous study of outdoor group walks [61]. 

##### Walk Characteristics

*Duration of WfH walk* was a computed variable based on the participant-provided start and finish times of the group walk. *Perceived walk intensity* was assessed by asking participants to “rate the physical intensity of the walk” on a single item, 10-point scale (0 = *very low*; 10 = *very high*). This scale was used in previous research of outdoor walking behaviour [88]. *Region* of England [89] in which the participant attended their WfH walk was also included; data obtained from the main study. 

### 2.4. Statistical Analysis

Pearson’s correlation coefficients for repeated measures using median scores [90] for all variables were performed (except duration, which being purely continuous, mean values were used). Multilevel modelling was used to further study the relationships within the data. As each participant completed a questionnaire for every group walk attended, we had multiple responses from each participant about their pre- and post-walk emotional well-being, environment type, indicators of perceived environmental quality, and walk characteristics. Multilevel modelling allowed us to include all available information in one model. Multilevel models are “regression models that allow the inclusion of both ‘fixed’ and ‘random’ effects” [91] (p. 1001). “Random effects” reflect the hierarchical structure of the data (questionnaires within participants); this improves the analysis by partitioning unexplained variation into systematic variation between respondents and residual variation between questionnaires within respondents. The predictor variables of interest are the “fixed effects”. Multilevel models have been used in previous analyses of Nature and health [3,35,92,93,94].

*Main effects and moderation.* Two separate models were fitted for each outcome variable. The first model analyzed the main (fixed) effects for each outcome variable. The second model explored the presence of interaction effects. All analyses were performed using the R software [95], with multilevel models being fitted with the *nlme* package [96].

For all main effects models, outcome variables were post-walk positive affect, post-walk negative affect and post-walk happiness. Predictor variables were region, environment type, perceived naturalness, perceived biodiversity (birds, butterflies, plants and trees), perceived restorativeness, duration of WfH walk, and perceived walk intensity. Pre-walk levels of positive affect, negative affect and happiness were included as a covariate. For implementation and interpretation purposes, those variables that could be directly interpretable in terms of a continuous or interval scale (*i.e.*, all variables except region, environment type and the three perceived biodiversity variables) were treated as continuous. For region, the reference category was London. The reference category for environment type was urban public space. Reference categories for the three biodiversity variables were: 0–4 types of birds; 0–4 types of butterflies; 0–9 types of plants and trees. Multicollinearity diagnostics for the predictor variables following Shieh and Fouladi [97] were conducted in order to determine which predictor variables should be included in the multilevel models. Residual plots from these models were analyzed to determine how closely these followed the normal distribution. Duration of WfH walk and perceived walk intensity were tested for a potential diminishing returns effect based upon previous research results [45]. No transformations were necessary for any of the outcome variables.

For all moderator models, *a priori* potential interactions were selected based on theory [53], as recommended by Wu and Zumbo [98]. Five interactions were analyzed in this study:

1. Perceived restorativeness and environment type.
2. Perceived restorativeness and perceived naturalness.
3. Perceived restorativeness and perceived bird biodiversity.
4. Perceived restorativeness and perceived butterfly biodiversity.
5. Perceived restorativeness and perceived plants and trees biodiversity.

Perceived restorativeness was the moderating variable. Predictor variables and the interaction term were mean-centered [98,99]. All five interactions for each outcome variable were tested. Only statistically significant interactions are reported. 

## 3. Results 

### 3.1. Descriptive Analysis

A total of 1009 questionnaires were returned during the 13-week study period by the 127 participants, resulting in a median of seven questionnaires per person (mode = 12) and a range of 1–32. Table 1 describes each environment type and the number of responses per category. Means and standard deviations of predictor and outcome variables are provided in Table 2. The mean was higher for post-walk positive affect (*M* = 36.28) and happiness (*M* = 7.95) when compared to the pre-walk values (*M* = 30.52; *M* = 6.45, respectively). There was a reduction in the average value for the negative affect after the walk (*M* = 10.53) compared to pre-walk values (*M* = 11.74).

**Table 1:** The frequency of different environment types in which participants walked during the 13-week study period (*n* = 1009 questionnaires).

Environment Type	Example Provided in Questionnaire	*Frequency* *n* (%)
Natural and semi-natural places	Country park, nature reserve	366 (36.3%)
Green corridor	River path, cycle ways, bridleways	206 (20.4%)
Urban green space ^a^	Public gardens, formal parks, amenity green space, allotments, community gardens, urban farms, outdoor sports pitches	195 (19.3%)
Farmland	*No example given*	127 (12.6%)
Urban public space	Streets, shopping centers, plaza	87 (8.6%)
Coastal	Seaside, estuary	15 (1.5%)
Mixture ^b^	*No example given*	11 (1.1%)

Note: Unable to be categorized *n* = 2. More detailed description of the environment types can be found in English Planning Policy Guidance 17 [100]. ^a^ = Author-created category combining: parks and gardens; allotments, community gardens and urban farms; amenity green space; and outdoor sports pitches. ^b^ = Category created from “other” write-in responses that described two or more different environment types.

### 3.2. Correlations

Table 2 shows the bivariate correlations. Post-walk positive affect was moderately correlated with pre-walk positive affect (*r* = 0.54, *p* < 0.001), perceived walk intensity (*r* = 0.38, *p* < 0.001) and perceived restorativeness (*r* = 0.60, *p* < 0.001). Similarly, post-walk happiness was moderately correlated with pre-walk happiness (*r* = 0.68, *p* < 0.001), perceived walk intensity (*r* = 0.45, *p* < 0.001) and perceived restorativeness (*r* = 0.43, *p* < 0.001). Post-walk negative affect was positively correlated with pre-walk negative affect only (*r* = 0.69, *p* < 0.001). Perceived naturalness, the three perceived biodiversity variables, and duration of WfH walk were not significantly correlated with any of the outcome variables.

**Table 2:** Correlations ^1^ between emotional well-being, perceived intensity, duration of WfH walk, and perceived biodiversity, naturalness and restorativeness.

Variables	M (SD)	Correlations between Variables											
		1	2	3	4	5	6	7	8	9	10	11	12
1. Pre-Walk Positive Affect	30.52 (7.32)	1											
2. Post-Walk Positive Affect	36.28 (6.99)	0.54 ^***^	1										
3. Pre-Walk Happiness	6.45 (2.10)	0.68 ^***^	0.26 ^**^	1									
4. Post-Walk Happiness	7.95 (1.50)	0.49 ^***^	0.62 ^***^	0.68 ^***^	1								
5. Pre-Walk Negative Affect	11.74 (3.63)	−0.16	0.11	−0.39 ^***^	−0.20	1							
6. Post-Walk Negative Affect	10.53 (1.71)	0.04	0.09	−0.144	−0.12	0.69 ^***^	1						
7. Perceived Naturalness	5.22 (1.58)	0.01	0.17	−0.02	0.08	−0.00	0.03	1					
8. Perceived Birds	NA	0.05	0.06	0.07	0.08	0.01	0.04	0.02	1				
9. Perceived Butterflies	NA	−0.01	0.07	0.03	0.09	0.08	−0.06	−0.02	0.55 ^***^	1			
10. Perceived Plants & Trees	NA	0.05	0.01	0.10	0.03	0.03	0.03	0.08	0.67 ^***^	0.46 ^***^	1		
11. Duration of WfH Walk	1.53 (0.97)	−0.04	0.10	−0.00	0.16	0.01	−0.06	0.15	0.20 ^*^	0.12	0.19 ^*^	1	
12. Perceived Walk Intensity	6.07 (1.81)	0.29 ^**^	0.38 ^***^	0.28 ^**^	0.45 ^***^	−0.09	0.03	0.30 ^**^	0.20 ^*^	0.17	0.15	0.11	1
13. Perceived Restorativeness	66.91 (15.11)	0.32 ^***^	0.60 ^***^	0.17	0.43 ^***^	0.01	−0.06	0.31 ^***^	0.34 ^***^	0.31 ^***^	0.25 ^**^	0.16	0.43 ^***^

Note: Environment type and region not included. Higher scores indicate greater: positive affect (range 10–50); negative affect (range 10–50); happiness (range 0–10), perceived naturalness (range 1–7); perceived birds (range 0–4 to 30+); perceived butterflies (range 0–4 to 20+) ; perceived plants and trees (range 0–9 to 300+); duration of WfH walks (range 0.25 to 6 h); perceived walk intensity (range 0–10), and perceived restorativeness (range 0–96). NA: not applicable. *n* = 127. ^*^ *p* < 0.05. ^**^ *p* < 0.01. ^***^ *p* < 0.001. ^1^ Pearson’s correlation coefficients for repeated measures calculated using median scores for all variables, except duration for which mean values were considered (relatively similar Spearman rank correlations were found).

### 3.3. Main Effects Multilevel Models

In this set of analyses, we studied the main effects of region, perceived environment type, indicators of perceived environmental quality, and walk characteristics on post-walk emotional well-being. 

#### 3.3.1. Positive Affect

Pre-walk positive affect (Coeff. = 0.441, *p* < 0.001), perceived restorativeness (Coeff. = 0.126, *p* < 0.001), and perceived walk intensity (Coeff. = 0.399, *p* < 0.001) were all significant predictors of post-walk positive affect (Table 3). Perceived restorativeness was a significant predictor of positive affect following an outdoor group walk, independent of the effect of perceived intensity. 

**Table 3:** Summary statistics for multilevel main effects model for post-walk positive affect (*n* = 935).

Random Effects				
Covariance Parameter		Covariance Estimate		
Individual		15.955		
Questionnaire		9.607		
**Fixed Effects (Type III)**				
**Variable**	**Coefficient**	**SE**	***F* Value**	***p***
Pre-walk Positive Affect	0.441	0.025	301.900	<0.0001
Region	--	--	1.102	0.367
Type of Environment	--	--	0.978	0.446
Perceived Naturalness	−0.178	0.105	2.852	0.092
Perceived Birds	--	--	1.499	0.213
Perceived Butterflies	--	--	1.461	0.224
Perceived Plants and Trees	--	--	0.830	0.477
Perceived Restorativeness	0.126	0.014	81.993	<0.0001
Duration of WfH Walk	−0.130	0.220	0.346	0.557
Perceived Walk Intensity	0.399	0.084	22.696	<0.0001

Note: The table does not show the effect estimates of categorical predictors. The model was fitted with an intercept.

#### 3.3.2. Happiness

Statistically significant main effects of post-walk happiness were found for pre-walk happiness (Coeff. = 0.358, *p* < 0.001), perceived restorativeness (Coeff. = 0.029, *p* < 0.001) and perceived walk intensity (Coeff. = 0.122, *p* < 0.001) (Table 4), indicating that as each predictor increased, post-walk happiness also increased. The effect of perceived restorativeness on post-walk happiness occurred after controlling for the effect of walk intensity. 

#### 3.3.3. Negative Affect

Pre-walk negative affect (Coeff. = 0.259, *p* < 0.001), perceived bird biodiversity (*p* = 0.008), and perceived restorativeness (Coeff. = −0.013), *p* = 0.009) all had significant main effects on negative affect (Table 5). Greater perceived restorativeness was associated with a reduction in post-walk negative affect. Examination of the differences in each bird biodiversity category found that post-walk negative affect significantly increased as the number of birds perceived during the walk increased from 0–4 to 5–14 species types (Coeff. = 0.444, SE = 0.144, *p* = 0.002). There were nonsignificant effects on post-walk negative affect from perceiving 15–30 (Coeff. = 0.136, SE = 0.231, *p* = 0.557) or more than 30 types of birds (Coeff. = 0.171, SE = 0.357, *p* = 0.631), when compared to 0–4 types of birds.

**Table 4:** Summary statistics for multilevel main effects model for post-walk happiness (*n* = 935).

Random Effects				
Covariance Parameter		Covariance Estimate		
Individual		0.465		
Questionnaire		0.653		
**Fixed Effects (Type III)**				
**Variable**	**Coefficient**	**SE**	***F* Value**	***p***
Pre-walk Happiness	0.358	0.020	318.700	<0.0001
Region	--	--	0.999	0.441
Type of Environment	--	--	1.627	0.124
Perceived Naturalness	−0.029	0.026	1.224	0.269
Perceived Birds	--	--	0.733	0.533
Perceived Butterflies	--	--	1.716	0.162
Perceived Plants and Trees	--	--	0.151	0.930
Perceived Restorativeness	0.029	0.003	76.146	<0.0001
Duration of WfH Walk	0.076	0.055	1.924	0.166
Perceived Walk Intensity	0.122	0.021	33.649	<0.0001

Note: The table does not show the effect estimates of categorical predictors. The model was fitted with an intercept.

**Table 5:** Summary statistics for multilevel main effects model for post-walk negative affect (*n* = 935).

Random Effects				
Covariance Parameter		Covariance Estimate		
Individual		0.251		
Questionnaire		2.026		
**Fixed Effects (Type III)**				
**Variable**	**Coefficient**	**SE**	***F* Value**	***p***
Pre-walk Negative Affect	0.259	0.015	293.829	<0.0001
Region	--	--	1.495	0.166
Type of Environment	--	--	0.652	0.713
Perceived Naturalness	0.020	0.042	0.221	0.639
Perceived Birds	--	--	3.967	0.008
Perceived Butterflies	--	--	1.018	0.384
Perceived Plants and Trees	--	--	1.468	0.222
Perceived Restorativeness	−0.013	0.005	6.805	0.009
Duration of WfH Walk	−0.123	0.085	2.081	0.150
Perceived Walk Intensity	0.044	0.033	1.795	0.181

Note: The table does not show the effect estimates of categorical predictors. The model was fitted with an intercept.

### 3.4. Moderation Multilevel Models

We also studied interaction effects to assess whether perceived restorativeness moderated the association between perceived environment type, naturalness or biodiversity, and emotional well-being. A significant interaction effect was found for positive affect only. No interaction effects were found for happiness or negative affect.

Table 6 shows the significant interaction model for positive affect. Of the five interaction models tested, the interaction of perceived restorativeness and perceived naturalness emerged as a significant predictor (Coeff. = 0.290, *p* = 0.027). The interaction indicates that the level of restorativeness moderated the association between perceived naturalness and positive affect. 

**Table 6:** Summary statistics for multilevel moderation model for post-walk positive affect (*n* = 935).

Random Effects				
Covariance Parameter		Covariance Estimate		
Individual		15.964		
Questionnaire		9.560		
**Fixed Effects (Type III)**				
**Variable**	**Coefficient**	**SE**	***F* Value**	***p***
Pre-walk Positive Affect	0.438	0.025	298.434	<0.0001
Region	--	--	1.102	0.367
Type of Environment	--	--	0.879	0.523
Perceived Naturalness	−0.230	0.166	1.912	0.167
Perceived Birds	--	--	1.399	0.242
Perceived Butterflies	--	--	1.470	0.221
Perceived Plants and Trees	--	--	0.834	0.475
Perceived Restorativeness	1.948	0.211	84.902	<0.0001
Duration of WfH Walk	−0.122	0.220	0.298	0.586
Perceived Walk Intensity	0.382	0.084	20.681	<0.0001
Perceived Restorativeness ^*^	0.290	0.131	4.913	0.027
Perceived Naturalness				

Note: Perceived naturalness, perceived restorativeness and the interaction term (^*^ perceived restorativeness and perceived naturalness) were mean centered. The table does not show the effect estimates of categorical predictors. The model was fitted with an intercept.

## 4. Discussion

This study explored the health benefits of Nature beyond a “green” environment to investigate the effects environment type and indicators of perceived environmental quality—naturalness, biodiversity and restorativeness—had on short-term emotional well-being following an outdoor group walk. Characteristics of the walk (*i.e.*, walk duration, intensity) were assessed to understand their independent relationship to emotional well-being. We also investigated whether perceived restorative quality moderated the effect of perceived environment type or perceived environmental quality on emotional well-being. 

We found that perceived restorative quality of the environment was a significant predictor of emotional well-being following a group walk, associated with an increase in positive affect and happiness as well as a reduction in negative affect. The identified relationship between perceived restorativeness and positive affect mirrors findings in research by Hartig *et al.* [7,55] and Sato [56]. To our knowledge, the influence of perceived restorativeness on negative affect is a unique finding. Further research is required to fully understand the effect of perceived restorativeness on negative affect. The type of environment and the other two indicators of perceived environmental quality—naturalness and biodiversity—were nonsignificant predictors of emotional well-being when combined in the same model with perceived restorative quality. These results suggest that restorative quality of an environment may be an important element for enhancing emotional well-being. 

Moreover, our analyses also showed that perceived restorative quality moderated the association between perceived naturalness and post-walk positive affect. In other words, perceived restorativeness and perceived naturalness interacted to enhance positive affect following an outdoor group walk. This finding suggests these two indicators may work together to amplify the experience of positive emotions in Nature. The significant interaction of perceived naturalness and restorative quality is supportive of ART, which considers natural environments to have plentiful restorative qualities [52,53]. Previous research has found that people differ in their assessments of an environment’s restorative quality based on its level of naturalness, in that more natural environments are rated as higher in perceived restorative quality than less natural environment [13,43,55,101,102,103,104]. To date no research has specifically investigated the interaction of the two on affective outcomes. Perhaps the closest study to ours was Gonzalez et al [64] who found the restorative quality ‘being away’ moderated the effect of a therapeutic horticulture intervention on depression [64]. It is important to note that Gonzalez *et al.* [64] captured the restorative experience of two environments (*i.e.*, home and the horticulture intervention setting) in their measure of being away. Thus to our knowledge, the interaction between perceived restorativeness and perceived naturalness on emotional well-being found here is novel. 

Taken together, these two results emphasize the importance of considering the transactional relationship between person and environment in Nature-health research. Perceived restorative quality is about a person’s experience of an environment as restorative, which is related to but separate from the other two indicators of perceived environmental quality examined in this study: naturalness and biodiversity. Our results suggest the short-term emotional well-being benefits of Nature may be a consequence of an individual’s experience of the physical environment as restorative, rather than the environment itself. These findings suggest a move away from a deterministic approach to environmental design or Nature and health research, in which the assumption that including a particular feature (e.g., water; variety of shrubs) into an environment or living in proximity to a certain environment type (e.g., coast) will result in greater emotional well-being. 

In terms of walk characteristics, perceived walk intensity was a significant predictor of greater positive affect and happiness following an outdoor group walk. The identified relationship between intensity and positive affect is consistent with previous research that found immediate gains in positive affect following objectively measured exercise intensity [69]. We found no significant predictive relationship of perceived intensity on post-walk negative affect. Duration of the group walks was not a significant predictor of emotional well-being. This finding differs from that of Ekkekakis [69] who identified a significant positive relationship between duration of physical exercise and post-exercise positive affect. Thus it may be that the perceived physical intensity of the walk contributes to greater positive emotional well-being following a group walk and that the duration of walking may not matter after accounting for intensity. The significance of physical intensity on post-walk positive emotional well-being highlights physical exercise as a possible confounder in green exercise studies. Moreover, the result calls attention to the need to isolate the effects of physical activity—such as walk intensity—from the effects of perceived environmental quality when investigating the health benefits of Nature [16,32,68]. This is especially important to tease out since providing a space for or enhancing the effects of physical activity has been suggested as one of the mechanisms through which Nature can affect human health [32].

Analyses also revealed perceived bird biodiversity was a significant predictor of post-walk negative affect. In contrast to previous research [19,41,42], negative affect increased as the perceived biodiversity of birds increased, specifically from 0–4 to 5–14 species types. The relationship could perhaps be explained by the type of bird species and its acoustic properties (pitch, intensity, roughness) as well as whether the increased number of birds was compatible with group walkers’ use of the environment [105]. For example, it is possible that participants made their assessment of perceived bird biodiversity based on bird song and calls, as this can be an one way in which to identify them [105]. In general, listening to birdsong improves mood [106]. However, not all bird sounds are perceived as positive, as the songs and calls of certain bird species common in English urban green spaces (e.g., magpies, crows, owls) are associated with negative emotions [105]. Perhaps the increased number of birds perceived by participants was one of these bird species, or that the additional bird species were incompatible to the participant’s use of the environment whilst on a group walk.

In this study, environment type, perceived biodiversity of butterflies, and plants and trees were not significant predictors of the change in emotional well-being following a group walk. Previous research [48] also found a nonsignificant effect of environment type for a group walk on positive affect. However, the authors did find a reduction in negative affect associated with group walks in farmland and green corridor environments. Methodological differences in the measurement of the emotional well-being between that study and ours make direct comparisons difficult; the previous study assessed longer-term emotional well-being associated with outdoor group walks in certain environments, whilst here we assessed short-term emotional well-being, *i.e.*, immediately following the walk. Three reasons for the nonsignificant effects of butterflies and plants and trees are discussed below. 

## 5. Conclusions

In summary, our findings indicate that perceived restorative quality and perceived walk intensity contributed to short-term emotional well-being. These findings extend current research on the effects of environment type [48], naturalness [50] biodiversity [19], and restorativeness [54] on well-being. Moreover, the finding that perceived restorative quality moderated the association between perceived naturalness and post-walk positive affect suggests that the two environmental quality indicators may amplify positive affective responses to Nature, and provides further insight into the transactional relationship between person and environment. The current findings add to a growing empirical literature documenting the health benefits of Nature [32,34]. The study highlights the contribution of perceived restorative quality to the relationship between environment type or indicators of environmental quality and well-being in Nature and health research. The study also further emphasises the need to control for effects of physical activity in green exercise research.

## 6. Limitations and Future Directions

This study has a number of limitations. First, the data may reflect a seasonal effect, as the study took place over the changing seasons from late summer through autumn. As changes in temperature and weather may influence response to perceived restorativeness and emotional well-being measures, future research may consider data collection during a single season. Second, it is beneficial to conduct a power analysis and estimate the required sample size prior to the data collection to ensure detection of moderation effects [107]. However, given the study design—in which the number of returned questionnaires was dependent on the number of group walks taken by participants during the study— an initial power analysis could not be performed. Third, low response frequencies for certain biodiversity categories might have increased the sensitivity of our models to external interactions; potential conclusions and inference will need to take this into account. Fourth, the data collection protocol could mean that participants did not necessarily complete the questionnaire immediately before and after the group walk, which could affect internal validity. Future smaller scale studies could place the experimenter with participants to ensure adherence to the data collection protocol. Finally, walking in a group may result in less interaction with the environment [108] and less perceived restorativeness [61]. As such, future studies may wish to replicate this study with solo walkers.

To our knowledge, the identified relationship between perceived biodiversity and emotional well-being following a group walk is novel. As research in perceived and objective biodiversity and subjective well-being is a nascent research area, we suggest our findings here be considered with caution until a greater evidence base is developed. We give three reasons for caution. First, over the course of the data collection (22 August to 14 November 2011), the number of actual species of birds and butterflies present in the walk setting likely diminished, and cues of different types of plants and trees may have become reduced as well. Second, the measures of perceived biodiversity used in this study were created specifically for investigating species richness in urban green spaces [40], but were applied here to assess perceived biodiversity in seven different environment types. As such, use of setting-specific perceived biodiversity response categories to assess perceived biodiversity in other environment types may be inappropriate. Finally, the perceived biodiversity measures were originally designed such that responses from participants could be compared to objective ecological survey data on species richness [40]. Consequently, these measures ask participants to make a numerical assessment, on a categorical response option scale, of the number of birds, butterflies and plants/trees in an environment. If researchers are not seeking to align measures of objective and subjective biodiversity, then future studies may want to use a more subjective scale of perceived biodiversity, like the Biodiversity Experience Index [41]. 

In this paper we specifically focused on how perceived restorativeness might interact with perceived environment type, naturalness and biodiversity to influence well-being. An alternative examination is to investigate perceived restorativeness as a mediator. Indeed, suggestive evidence of mediation appears in the reported bivariate correlations; measures of perceived naturalness and biodiversity did not correlate with post-walk emotional well-being but did significantly correlate with perceived restorativeness, which was significantly correlated with the post-walk emotional well-being. Thus, perceived naturalness and biodiversity may *indirectly* influence post-walk emotional well-being *via* perceived restorativeness. Future studies could usefully investigate a mediation model in which perceived restorativeness mediates the relationship between environment type, naturalness and biodiversity on well-being [109]. We are currently reanalysing our data to explore whether such a multilevel mediation model exists.

Environmental experiences are multi-dimensional as environmental types and qualities co-occur. As such, future studies on Nature and health could usefully investigate the interaction of environment type and/or indicators of perceived environmental quality on health and well-being outcomes. Further research is required to determine whether perceived restorative quality moderates the Nature-health relationship. 
